# 74 Understanding the Effect of Health Literacy on Burn Outcomes

**DOI:** 10.1093/jbcr/iraf019.074

**Published:** 2025-04-01

**Authors:** Jessica Schucht, Mina Nordness, Robyn Tamboli, Sunil Kripalani, Jesse Wrenn, Anne Wagner, Elizabeth Slater, Stephen Gondek, Bradley Dennis, Robel Beyene

**Affiliations:** Vanderbilt Burn Center; Vanderbilt Burn Center; Vanderbilt Burn Center; Vanderbilt University Medical Center; Vanderbilt Emergency Medicine; Vanderbilt University Medical Center; Vanderbilt University Medical Center; Vanderbilt University Medical Center; Vanderbilt University Medical Center; Vanderbilt University Medical Center

## Abstract

**Introduction:**

Health literacy can be a key factor in patient outcomes and self-management, yet the overall role of health literacy in burn outcomes is not well known. Understanding the effects of health literacy on burn patients can allow us to better care for them and anticipate needs and boundaries for a safe disposition. Our burn center aims to perform routine health literacy screening for all patients. This study examines the health literacy of burn survivors and seeks to determine its effect on unplanned emergency room visits within 30 days of discharge.

**Methods:**

Single-center, retrospective review of all patients admitted to the burn service between 2018-2024 who completed the Brief Health Literacy Screen (BHLS) at admission. BHLS scores were divided into low (< 9), moderate (9-11) and high (>11). The burn registry was queried for those who returned to the emergency department within 30 days of original disposition. For simplicity, disposition was divided into four categories: home without services, home with services, discharged to inpatient facility with wound care services, or discharged to a location other than home without wound care services (jail, unhoused, inpatient psychiatric facility, against medical advice). Multiple logistic regression was performed to determine the effect of BHLS on unplanned returns to ED. Covariates included total body surface area, LOS, Charleson Comorbidity Index (CCI), and disposition.

**Results:**

After eliminating patients < 18yo, in hospital mortalities, and those with missing data or BHLS screens, our study group included 1079 patients. Of these patients, 7% (n=77) had unplanned return visits to the emergency department within 30 days of discharge. 156 (14.5%) patients had low health literacy scores, 271 (25.1%) had moderate scores, and 652 (60.4%) had high scores. When controlling for other factors, health literacy did not affect return to the ED within 30 days of discharge. Interestingly, disposition to a location other than home without structured wound care in place significantly increased the likelihood of an unplanned return to the ED at 30 days (OR 8.24, 95% CI 3.11-21.84, p< 0.05).

**Conclusions:**

Contrary to our initial hypothesis, low BHLS scores were not associated with return to the emergency department within 30 days of discharge. However, discharge anywhere except home without structured wound care services was found to increase the likelihood of return to ED. As many of our patients fall into this category, further research could elucidate ways to improve disposition planning within this subset of patients. Finally, analysis was limited by the fact that only 36% of burn patients were screened in the study timeframe, well below hospital average of 71%.

**Applicability of Research to Practice:**

Understanding the factors that lead to unplanned return visits to the ED can help us better care for our patients and prepare them for discharge.

**Funding for the Study:**

N/A

